# The impact of prostate size on outcomes of aquablation: a prospective study

**DOI:** 10.1007/s00345-026-06588-7

**Published:** 2026-07-30

**Authors:** Dolev Perez, Ariel Mamber, Michael Pasherstnik, Dmitry Koulikov, Ala Eddin Natsheh, Ofer Z. Shenfeld, Ilan Z. Kafka, Boris Chertin

**Affiliations:** The Department of Urology, Faculty of Medicine, Shaare Zedek Medical Center, Hebrew University, P.O.B. 3235, 91031 Jerusalem, Israel

**Keywords:** Aquablation, Benign prostatic hyperplasia, Prostate size, Functional outcomes

## Abstract

**Purpose:**

To evaluate whether prostate size influences perioperative, urinary, and sexual outcomes following Aquablation for benign prostatic hyperplasia (BPH), by comparing men with prostates < 80 cc and ≥ 80 cc treated within a prospective real-world cohort.

**Methods:**

This prospective, single-center cohort study included consecutive men undergoing Aquablation between 2023 and 2025. Patients were stratified by prostate volume (< 80 cc vs. ≥80 cc). Baseline characteristics, perioperative parameters, and functional outcomes were assessed using validated instruments, including IPSS, IPSS-QoL, irritative subscore, ICIQ-SF, IIEF-EF, MSHQ-EjD, and MSHQ-bother, at baseline and at 3, 6, 12, and 24 months. Complications were graded according to the Clavien–Dindo classification.

**Results:**

A total of 628 men were included (324 with prostates < 80 cc and 304 with prostates ≥ 80 cc). Men with larger prostates had higher rates of preoperative catheter dependence (34% vs. 18.2%, *p* < 0.001). Operative time (40.6 ± 14.1 vs. 36.2 ± 12.6 min, *p* = 0.001) and hospitalization duration (2.5 ± 1.5 vs. 2.09 ± 0.8 days, *p* < 0.001) were longer in the ≥ 80 cc group. Both cohorts demonstrated marked and durable improvement in urinary symptoms, with mean IPSS improving from 24.5 to 6.7 in the < 80 cc group and from 24.9 to 6.0 in the ≥ 80 cc group at 24 months. Irritative symptom scores improved from 10.3 to 3.5 and from 10.5 to 2.1, respectively (*p* < 0.001). Sexual outcomes remained stable across prostate sizes, with preserved erectile function and low ejaculatory bother scores. High-grade complications (Clavien–Dindo III–IV) were uncommon and comparable between groups (2.7% vs. 4.3%), while reoperation rates remained low (3.1% vs. 3.9%).

**Conclusion:**

Aquablation provides durable symptom relief with preservation of sexual function across a broad range of prostate volumes, supporting its role as a size-independent surgical treatment option for BPH.

**Supplementary Information:**

The online version contains supplementary material available at 10.1007/s00345-026-06588-7.

## Introduction

Benign prostatic hyperplasia (BPH) is one of the most common urologic conditions in aging men, with a prevalence approaching 70% in the seventh decade and nearly 80% in men older than 70 years [1]. Progressive prostate enlargement may lead to moderate or severe lower urinary tract symptoms (LUTS), significantly impairing quality of life and contributing to complications such as acute urinary retention, recurrent urinary tract infections, and renal dysfunction [2, 3]. Transurethral resection of the prostate (TURP) remains the traditional gold standard surgical treatment for prostates between 30 and 80cc [4]. However, its well-recognized morbidity profile, including risks of bleeding, transfusion, urethral stricture, and sexual or ejaculatory dysfunction, has stimulated the adoption of minimally invasive surgical therapies (MISTs) aimed at reducing perioperative complications while maintaining clinical efficacy [5].

Aquablation is a robotic, image-guided waterjet ablation technology designed to resect prostatic tissue under real-time ultrasound guidance while minimizing thermal injury and preserving sexual and ejaculatory function [6]. Clinical trials and real-world series have demonstrated significant and durable symptom improvement, with favorable safety outcomes, particularly in prostates measuring 30–80 cc, the range recommended by the American Urological Association (AUA) and European Association of Urology guidelines recommendation [7–9].

For large prostates (≥ 80–100 cc), standard surgical options include Holmium Laser Enucleation of the Prostate (HoLEP), Thulium Laser Enucleation (ThuLEP), and simple prostatectomy [9]. Although they are highly effective, these procedures are associated with longer operative time, increased perioperative resource utilization, and a substantial learning curve [10].

At the same time, minimally invasive surgical therapies (MISTs) are increasingly being expanded to larger prostates. Recently, the WATER III trial showed noninferior short-term symptom relief, superior preservation of ejaculatory function, and lower rates of persistent stress urinary incontinence with Aquabltion compared with laser enucleation in large prostates [11].

Despite the evidence that supports Aquabltion on glands up to 150 cc> [12], comparative data evaluating the impact of prostate size on perioperative outcomes and urinary and sexual functions remain limited.

Therefore, the present study aimed to compare perioperative characteristics, functional outcomes, sexual and ejaculatory function, and complication rates between men undergoing Aquablation with prostates < 80 cc and ≥ 80 cc.

## Methods and materials

This prospective, single-center cohort study included consecutive men undergoing Aquablation for symptomatic benign prostatic hyperplasia between January 2023 and December 2025. Patients were stratified post hoc according to prostate volume measured by transrectal ultrasound (< 80 cc vs. ≥80 cc), a clinically established threshold consistent with American Urological Association guideline recommendations and the volume stratification used in the pivotal WATER trials [2, 7]. The study was approved by the institutional Ethics Committee of Shaare Zedek Medical Center (approval number: SZMC-0058-25).

All procedures were performed using the standard Aquablation technique, including targeted resection under real-time ultrasound guidance, followed by selective hemostasis with focal coagulation at the bladder neck when indicated. All surgeries were performed by a single experienced surgeon (BC) or under his direct supervision, ensuring procedural consistency throughout the study period. Postoperative management was standardized across the study cohort. Urethral catheters were generally removed approximately 48 h after surgery, depending on the degree of hematuria and clinical recovery. Anticoagulant and antiplatelet therapy was managed perioperatively in consultation with the patient’s treating specialist and was resumed following adequate resolution of hematuria. In patients considered at high thromboembolic risk, temporary bridging therapy with low-molecular-weight heparin was administered before resumption of chronic anticoagulation.

Baseline data included age, prostate-specific antigen (PSA), prostate volume, presence of median lobe, preoperative catheter dependence, prior prostate surgery, antithrombotic therapy, and BPH medication use. Perioperative variables included the American Society of Anesthesiologists (ASA) score, operative time (from handpiece insertion to catheter placement), length of hospitalization, and bladder stone treatment when indicated.

Functional outcomes were assessed using validated questionnaires, including IPSS, IPSS quality-of-life index, irritative subscore, visual analog scale (VAS) and ICIQ-SF. Sexual function was assessed using the IIEF-EF, MSHQ-EjD, and MSHQ bother score, which were administered only to sexually active patients.

Follow-up data were available for 321 patients at 3 months, 296 at 6 months, 259 at 12 months, and 236 at 24 months in the < 80 cc cohort (*n* = 324). In the ≥ 80 cc cohort (*n* = 304), follow-up data were available for 283 patients at 3 months, 269 at 6 months, 240 at 12 months, and 210 at 24 months. All longitudinal analyses were performed according to available follow-up data at each time point.

Statistical analysis was performed using GraphPad Prism version 9. Continuous variables were compared using Student’s t-test or Mann–Whitney U test, as appropriate, and categorical variables using Fisher’s exact test. Longitudinal within-group comparisons were analyzed using one-way ANOVA. All tests were two-sided, with *p* < 0.05 considered statistically significant.

## Results

A total of 628 men underwent Aquablation, including 324 with prostates < 80 cc and 304 with prostates ≥ 80 cc. Baseline characteristics differed between groups, with higher PSA levels, larger prostate volumes, more frequent median lobe enlargement, and higher rates of preoperative indwelling catheterization in the ≥ 80 cc cohort (all *p* < 0.05), while age, prior prostate surgery, and antithrombotic use were comparable (Table 1).

**Table 1:** Baseline demographic and clinical characteristics (Continuous variables are presented as mean ± standard deviation)

	Prostate < 80 cc (*n* = 324)	Prostate > 80 cc (*n* = 304)	*P* value
Age, years	70.5 ± 8.5	72.2 ± 7.8	***0.008***
PSA, ng/mL	3 ± 2.3	5.9 ± 1.5	***< 0.001***
Prostate volume, cc	60.6 ± 13.3	117.6 ± 34.6	***< 0.001***
Median lobe present, n (%)	120 (37.0%)	138 (45.3%)	***0.039***
Pre-procedure indwelling catheterization n (%)	59 (18.2%)	103 (34%)	***< 0.001***
Prior prostate surgery n (%)	11 (3.4%)	11 (3.6%)	0.89
Antithrombotic use n (%) Anticoagulation Antiplatelet Any of above			
	39 (12.2%)	35 (11.5%)	0.80
	104 (31.9%)	94 (30.9%)	0.79
	143 (44.1%)	129 (42.4%)	0.67
BPH medication use n (%) Alpha blocker 5-ARI Alpha blocker/5-ARI Any of above			
	205 (63.2%)	222 (73%)	***< 0.009***
	16 (4.9%)	14 (4.6%)	0.086
	62 (19.1%)	52 (17.1%)	0.53
	283 (87.2%)	288 (94.7%)	***0.001***

Bold and italics indicate statistically significant results (p<0.05).

Perioperative outcomes demonstrated longer operative time and hospitalization in the ≥ 80 cc group (*p* = 0.001 and *p* < 0.001, respectively), with similar ASA score distributions between cohorts (Table 2).

**Table 2:** Perioperative characteristics and postoperative complications

	Prostate < 80 cc (*n* = 324)	Prostate > 80 cc (*n* = 304)	*P* value
ASA score, *n* (%) Grade I Grade II Grade III Grade IV			0.867
	5 (1.5%)	6 (1.9%)	0.77
	232 (71.6%)	223 (73.3%)	0.63
	85 (26.2%)	74 (24.3%)	0.56
	2 (0.6%)	1 (0.3%)	0.62
Operative time (handpiece in to catheter in), min	36.2 ± 12.6	40.6 ± 14.1	***0.001***
Hospitalization length, days	2.09 ± 0.8	2.5 ± 1.5	***< 0.001***
Need for postoperative BPH medication, n (%)	8 (2.5%)	15 (4.9%)	0.136
Re- operation, n(%)	10 (3.1%)	12 (3.9%)	0.666
Clavien- Dindo classification Grade I Grade II Grade III Grade IV			
	22 (6.7%)	18 (5.9%)	0.74
	28 (8.6%)	45 (14.8%)	*0.018*
	8 (2.4%)	9 (2.9%)	0.81
	1 (0.3%)	4 (1.3%)	0.20

Bold and italics indicate statistically significant results (p<0.05).

**Table 3:** Urinary functional outcomes over follow-up Values marked with * indicate statistically significant differences versus baseline within the same group (*p* < 0.05)

Parameter	Timepoint	Prostate < 80 cc (*n* = 324)	Prostate > 80 cc (*n* = 304)	*P* value
IPSS	Baseline 3 months 6 months 12 months 24 months	24.5 ± 6.0 7.1 ± 5.4* 5.6 ± 5.9* 6.2 ± 5.0* 6.7 ± 5.2*	24.9 ± 6.1 6.5 ± 5.8* 4.9 ± 4.7* 5.4 ± 5.2* 6 ± 5.1*	0.41 0.17 0.09 ***0.048*** 0.10
IPSS QoL	Baseline 3 months 6 months 12 months 24 months	5.3 ± 1.2 1.3 ± 1.4* 1.4 ± 2.8* 1.1 ± 1.5* 1 ± 1.5*	5.1 ± 1.3 1.1 ± 1.4* 0.8 ± 1.1* 1.1 ± 1.5* 0.8 ± 1.4*	***0.046*** 0.074 ***0.003*** 1 ***0.064***
Irritative score	Baseline 3 months 6 months 12 months 24 months	10.3 ± 3.6 3.4 ± 4.0* 2.5 ± 3.5* 3.7 ± 5.1* 3.5 ± 4.7*	10.5 ± 3.7 2.2 ± 2.6* 2 ± 2.8* 2.3 ± 2.8* 2.1 ± 2.0*	0.493 ***< 0.001*** ***0.048*** ***< 0.001*** ***< 0.001***
VAS score	Baseline 3 months 6 months 12 months 24 months	0 0.47 ± 1.9 0.42 ± 2.0 0.15 ± 0.4 0	0 0.34 ± 1.1 0.2 ± 1.5 0.1 ± 0.3 0	1 0.29 0.12 0.075 1
ICIQ-SF Score	Baseline 3 months 6 months 12 months 24 months	14.5 ± 5.0 11.9 ± 4.4* 13.2 ± 3.9 9.5 ± 6.0 8.7 ± 4.0*	14.1 ± 4.1 10.5 ± 5.4* 10.5 ± 4.2* 9 ± 4.3* 8.1 ± 4.0*	0.27 ***< 0.001*** ***< 0.001*** 0.23 0.061

Bold and italics indicate statistically significant results (p<0.05).

Significant improvements in urinary symptoms were observed in both groups across all follow-up intervals. Mean IPSS improved from 24.5 ± 6.0 to 6.7 ± 5.2 in the < 80 cc group and from 24.9 ± 6.1 to 6.0 ± 5.1 in the ≥ 80 cc group at 24 months. Similarly, IPSS-QoL scores improved from 5.3 ± 1.2 to 1.0 ± 1.5 and from 5.1 ± 1.3 to 0.8 ± 1.4, respectively. Irritative subscores tended to be lower in the ≥ 80 cc cohort at the first, second, third, and 24-month follow-up visits (all *p* ≤ 0.048). Urinary continence outcomes assessed by ICIQ-SF improved in both cohorts, with mean scores decreasing from 14.5 ± 5.0 to 8.7 ± 4.0 in the < 80 cc group and from 14.1 ± 4.1 to 8.1 ± 4.0 in the ≥ 80 cc group at 24 months (Table 3).

Sexual function remained largely stable over time in both groups. IIEF-EF scores demonstrated minor fluctuations with statistically significant differences at isolated intervals but without clinically relevant deterioration. Ejaculatory function, as measured by MSHQ-EjD, declined postoperatively in both cohorts, while associated bother scores remained low and stable throughout follow-up (Table 4).

**Table 4:** Sexual and ejaculatory functional outcomes over follow-up Values marked with * indicate statistically significant differences versus baseline within the same group (*p* < 0.05)

Parameter	Timepoint	Prostate < 80 cc (Sexually active patients, *n* = 198)	Prostate > 80 cc (Sexually active patients, *n* = 201)	*P*- value
EF-IIEF	Baseline 3 months 6 months 12 months 24 months	20.1 ± 9.2 18.7 ± 9.4 17.7 ± 10.8* 17.5 ± 11* 17.9 ± 10	24.9 ± 6.1 18.4 ± 9.9 19 ± 9.6 19.5 ± 8.9 19 ± 8	***< 0.001*** 0.74 0.18 ***0.041*** 0.27
MSHQ-EjD	Baseline 3 months 6 months 12 months 24 months	10.4 ± 4.8 7.3 ± 5.8* 6.7 ± 5.1* 6.7 ± 6.4* 6.1 ± 5*	9.6 ± 4.2 8.2(± 5 8 ± 5.8* 8.9 ± 5.1 8 ± 4.2*	***0.0.26*** ***0.037*** ***0.0015*** ***< 0.001*** ***< 0.001***
MSHQ-bother score	Baseline 3 months 6 months 12 months 24 months	1.1 ± 1.6 2.2 ± 2.4 2.1 ± 2.1 1.9 ± 2.8 1.2 ± 1.6	1.3 ± 1.6 1.6 ± 1.7 1.6 ± 1.6 1.1 ± 1.3 1 ± 1.7	0.18 ***0.004*** ***0.007*** ***< 0.001*** 0.29

Bold and italics indicate statistically significant results (p<0.05).

Retreatment rates were low and comparable between groups. Among men with prostates < 80 cc, 8 patients (2.5%) initiated BPH medication and 10 (3.1%) required reoperation at a mean of 8.1 months. In the ≥ 80 cc cohort, 15 patients (4.9%) initiated medication and 12 (3.9%) underwent surgical retreatment at a mean of 4.2 months. Of these, five patients underwent repeat surgical intervention immediately or shortly after the index procedure, reflecting early cases during the initial learning curve of Aquablation implementation. No significant differences were observed in medication use (*p* = 0.136) or reoperation rates (*p* = 0.666).

Postoperative complication profiles are summarized in Table 2. Overall complication rates were comparable between cohorts. Minor complications (Clavien–Dindo Grade I) occurred at similar frequencies, whereas Grade II complications, including urinary retention, urinary tract infection, and blood transfusion, were more common in the > 80 cc group. Grade III complications were primarily related to postoperative hematuria requiring endoscopic intervention, including clot evacuation and fulguration. Grade IV events were rare in both groups. Importantly, rates of higher-grade complications (Clavien–Dindo Grades III–IV) were low and did not differ significantly between prostate-size groups.

## Discussion

Aquablation has become a widely adopted minimally invasive surgical treatment for benign prostatic hyperplasia (BPH), with increasing evidence supporting its effectiveness across various prostate sizes [7]. However, most high-quality evidence on Aquablation has come from studies evaluating moderate and large prostates separately, limiting direct insight into how prostate volume affects outcomes across the full disease spectrum [6]. In this real-world, size-stratified analysis, Aquablation demonstrated sustained improvements in urinary symptoms, stable sexual function, and low retreatment rates in both moderate and large prostates, despite a significantly higher baseline disease burden in the larger group.

Men with prostates ≥ 80 cc in our cohort presented with more advanced disease, including larger gland volumes, more frequent median lobe enlargement, and significantly higher rates of preoperative catheter dependence. Such characteristics have traditionally been associated with inferior outcomes following TURP and increased perioperative morbidity, particularly in larger glands [5, 13]. Prior TURP series have consistently demonstrated longer operative times, higher bleeding risk, and increasing retreatment rates with increasing prostate size, prompting guideline recommendations to limit TURP primarily to prostates ≤ 80 cc [13, 14]. In contrast, our findings demonstrate that Aquablation achieves comparable improvements in IPSS, quality of life, and irritative symptoms regardless of gland size, suggesting that its efficacy is not constrained by prostate volume as observed with conventional resection techniques [14, 15]. These observations are consistent with our previously published prospective comparative analysis of Aquablation, TURP, and HoLEP, in which Aquablation achieved greater symptom improvement despite treating significantly larger prostates, further supporting its volume-independent therapeutic performance [16].

When contextualized with pivotal trial data, our real-world results closely match those of the WATER and WATER II studies. The WATER trial established Aquablation as non-inferior to TURP for moderate-sized prostates (30–80 cc) [6], while WATER II extended these findings to large prostates (80–150 cc), showing sustained symptom relief and low retreatment rates at mid-term follow-up [15]. Importantly, these trials evaluated size categories independently. By directly comparing moderate and large prostates within a single cohort while all surgeries were performed by a single surgeon, our study builds on these findings. It provides evidence that Aquablation delivers consistent results across prostate sizes in routine clinical practice. This helps bridge the gap between randomized trials and real-world experience.

Notably, men with larger prostates in our cohort showed equal or greater reductions in irritative symptoms at several follow-up points. This contrasts with outcomes reported after enucleation-based procedures like HoLEP, where postoperative irritative symptoms and transient stress urinary incontinence are relatively common, especially in the early postoperative period [10, 17]. Published HoLEP series report early stress urinary incontinence rates ranging from 7% to 15%, with gradual recovery over several months [17–19], as well as nearly universal loss of antegrade ejaculation [19, 20]. While HoLEP remains highly effective for debulking large adenomas and provides excellent long-term symptom relief, its functional morbidity and steep learning curve are essential considerations [21]. In this context, our findings suggest that Aquablation may offer a favorable balance between effective relief of obstruction and preservation of function, especially in patients with large glands who prioritize postoperative continence and sexual function.

Perioperative outcomes in our study demonstrated expected volume-related differences, including modestly longer operative times and hospital stays in men with larger prostates. Similar trends have been reported in multicenter Aquablation registries and real-world analyses [22, 23], as well as in comparative studies of HoLEP and simple prostatectomy [24]. Importantly, these differences did not translate into higher rates of major complications. High-grade (Clavien–Dindo III–IV) events were rare and comparable between groups, while the observed increase in Grade II complications in larger prostates likely reflects greater baseline catheter dependence and postoperative medical management rather than procedure-specific morbidity [9, 22]. These findings compare favorably with open and robotic simple prostatectomy, which, although effective for very large glands, are associated with longer hospitalization, higher transfusion rates, and greater overall invasiveness [25].

Importantly, there is growing recognition that Aquablation may not fully align with the traditional definition of a minimally invasive surgical therapy (MIST). Recently published Delphi consensus data demonstrated a lack of expert agreement regarding its classification as a true MIST, largely due to its tissue-resecting mechanism, perioperative characteristics, and applicability to larger prostates [26].

Sexual functional outcomes are a key factor distinguishing modern BPH surgical treatments. In our cohort, erectile function remained stable across different prostate sizes, and ejaculatory bother scores remained low throughout follow-up, despite some decline in ejaculatory function scores. These results align with randomized studies showing significantly lower rates of retrograde ejaculation and better preservation of antegrade ejaculation with Aquablation compared to TURP and conventional laser enucleation methods [27, 28]. Importantly, in our previously published prospective comparative cohort, Aquablation preserved antegrade ejaculation in over 80% of sexually active men, significantly outperforming both TURP and HoLEP, findings that are reinforced by the current size-stratified analysis [17]. Propensity score analyses have also indicated better ejaculation preservation with Aquablation versus HoLEP [29]. Since preserving ejaculation is increasingly regarded as an important priority for many men undergoing BPH surgery, the consistent sexual outcomes seen in both moderate and large prostates provide a meaningful clinical advantage.

This study is strengthened by a large real-world cohort, standardized follow-up intervals, and the use of validated urinary and sexual outcome measures. Limitations include a single-center design and surgeries performed by a single experienced surgeon, which may limit reproducibility and generalizability. In addition, baseline clinical characteristics differed between the study groups, reflecting the higher burden among men with larger prostates. Other limitations include the absence of a direct surgical comparator, follow-up limited to two years, and incomplete characterization of anatomical factors such as intravesical prostatic protrusion or transition-zone volume. Future studies with longer-term follow-up and head-to-head comparisons with enucleation-based procedures are warranted to further define Aquablation’s role in the management of large and anatomically complex prostates.

## Conclusions

In this real-world, size-stratified cohort, Aquablation provided durable improvement in lower urinary tract symptoms with preservation of urinary continence and sexual function across a broad range of prostate volumes. Despite greater baseline disease severity in men with larger prostates, perioperative safety, functional outcomes, and retreatment rates were comparable to those observed in men with smaller prostates. These findings support Aquablation as a scalable, size-independent surgical option for benign prostatic hyperplasia, enabling effective symptom relief while maintaining key functional outcomes, even in large and anatomically complex prostates.

## Supplementary Information

Below is the link to the electronic supplementary material.

Supplementary Material 1

## Data Availability

No datasets were generated or analysed during the current study.
